# Clinical usefulness of high levels of C-reactive protein for diagnosing epithelial ovarian cancer

**DOI:** 10.1038/s41598-020-77167-y

**Published:** 2020-11-18

**Authors:** Desheng Yang, Haini Li, Xiaoyan Sun, Shengmei Yang, Kewei Wang, Zongtao Liu

**Affiliations:** 1 Department of Clinical Laboratory, Qingdao Hospital of Traditional Chinese Medicine (Qingdao Hiser Hospital), Qingdao, 266011 China; 2 Department of Gastroenterology, Qingdao Sixth People’s Hospital, Qingdao, 266001 China; 3 Department of Gynecology, Affiliated Qingdao Third People’s Hospital, Qingdao University, Qingdao, 266021 China; 4 Department of Gynecology, Qingdao University Affiliated Hospital, Qingdao, 266001 China; 5 Department of Pharmacology, Qingdao University School of Pharmacy, Qingdao, 266021 China; 6 Department of Clinical Laboratory, Affiliated Qingdao Third People’s Hospital, Qingdao University, Qingdao, 266021 China

**Keywords:** Cancer, Biomarkers, Medical research, Risk factors

## Abstract

The purpose of the present study was to evaluate the diagnostic role of CRP in ovarian cancer and to assess whether CRP can be combined with tumor markers to enhance the diagnostic efficacy toward ovarian cancer. Area under the curve, sensitivity, and specificity were calculated to access the diagnostic ability of each singly and combined as markers for ovarian cancer. The CRP cut-off value was then calculated to evaluate the diagnostic efficacy of CRP for ovarian cancer. Our results showed that values for all markers were significantly higher in the cancer group than in the control group. Receiver operating characteristic curve results showed that CA125 had the highest diagnostic efficacy for ovarian cancer, while the sensitivity for CRP was higher than for CA125, and the specificity for CRP was equal to that of CA125. The combination of CRP, CA125, and HE4, however, provided the strongest diagnostic capability. Furthermore, the diagnostic cut-off value for CRP with regard to ovarian cancer was 9.8 mg/L, and high levels of CRP were correlated with stage and tumor size of ovarian cancer. Our study indicated that CRP is valuable in the diagnosis of ovarian cancer, and that combining CRP with CA125 and HE4 improved the diagnostic efficacy with respect to ovarian cancer.

## Introduction

Ovarian cancer is one of the most common gynecologic malignancies and a leading cause of death in women^1,2^. Most ovarian cancers are epithelial ovarian cancers (EOC), and there are 4 pathologic types: serous, endometrioid, mucinous, and clear-cell ovarian cancer. Serous ovarian cancer is the most common, accounting for 40% of all epithelial tumors^3,4^. Early detection of EOC has a good prognosis, although the 5-year survival rate for patients diagnosed with advanced EOC is very low (20–40%)^5,6^. The initial signs and symptoms of EOC are not obvious, and the early diagnosis rate of EOC is only 20%^6^. Therefore, the principal strategy for controlling EOC is to develop effective methods with high specificity and sensitivity in cancer detection.

Serum cancer antigen 125 (CA125) and transvaginal ultrasonography are used clinically to screen for EOC^7^. However, CA125 is often elevated in benign conditions such as endometriosis and ovarian cysts, and is not always detectable in early-stage disease^8–11^—thus limiting CA125′s sensitivity and specificity for early detection. Human epididymis secretory protein E4 (HE4)—another biomarker for the diagnosis of EOC—exhibits similar limitations in detecting early and asymptomatic cancers^12,13^.

C-reactive protein (CRP) is a non-specific biologic marker of systemic inflammation that is released by hepatocytes in response to tissue injury and inflammation^14–16^. High levels of CRP have been associated with the risk for several chronic conditions, including cardiovascular disease, atherosclerosis, and cancer^15,17–19^. Investigators have reported that plasma CRP concentrations are increased in response to inflammation, which may promote tumor growth and metastasis in breast cancer and ovarian cancer^15,20,21^. However, additional clinical evidence is needed to prove a diagnostic role for CRP in EOC.

In the present study, we showed that the diagnostic capability of CRP for EOC is slightly weaker than that for CA125, but stronger than that for HE4. However, when we combined CRP, CA125, and HE4, we noted an improvement in the ability to diagnose EOC. Serum CRP levels were also correlated with tumor size and stage of EOC. The aforementioned results indicated that CRP is of great value in the diagnosis of EOC.

## Results

### EOC is correlated with age and parity—but not with BMI, smoking, or menopausal status

In Table 1, we summarized the baseline characteristics of the control group and patients with epithelial ovarian cancers according to the analysis of different groups of people, and found that the mean age at enrollment in the healthy control group, benign tumor group, stage I/II EOC group, and stage III/IV EOC group was 60.7, 59.7, 62.3, and 63.6 years, respectively. The mean parity in the same groups was 1.92, 1.84, 1.59, and 1.62, respectively, and the mean body mass index (BMI) was 25.4, 25.5, 25.6, and 25.5 kg/m^2^, respectively. Among the clinical parameters of the control and case groups, EOC was correlated with age and parity, but was not related to body mass index, menopausal status, or smoking status. Our results indicated that older women and women who had fewer children were at higher risk of EOC.

**Table 1:** Baseline characteristics of the control group and patients with epithelial ovarian cancers.

	Control group (n = 81 )		EOC group (n = 91)		*p*
	Healthy (n = 24)	Benign (n = 57)	Stage I/II (n = 52)	Stage III/IV (n = 39 )	
Age at enrollment, mean (SD)	60.7 (52–72)	59.7 (42–71)	62.3 (49–77)	63.6 (54–76)	< 0.05*^1^
Parity	1.92 (1–3)	1.84 (1–3)	1.59 (1–4)	1.62 (0–3)	< 0.05*^3^
**Smoking status**					0.79^2^
Yes (%)	9 (37.5)	18 (31.6)	21 (40.4)	13 (33.3)	
No (%)	15 (62.5)	39 (68.4)	31 (59.6)	26 (66.7)	
BMI	25.4 (24.2–26.3)	25.5 (24.1–26.6)	25.6 (24.7–26.3)	25.5 (24.1–26.5)	0.89^1^
**Menopausal status**					0.84^2^
Premenopausal (%)	10 (41.6)	24 (42.1)	18 (34.6)	14 (35.8)	
Postmenopausal (%)	14 (58.4)	33 (57.9)	34 (65.4)	25 (64.2)	
CRP, mean, (range) (mg/L)	2.8 (0.8–7.3)	4.5 (1.2–14)	14.4(1.6–35)	20.3 (1.8–54)	< 0.05*^3^
CA125, mean, (range) (U/ml)	11.5 (3.5–42)	21.9 (3.4–122)	126 (7.8–427)	162.8 (13.6–691)	< 0.05*^3^
HE4, mean, (range) (pmol/L)	53.2 (5.1–252)	75.5 (1.5–442)	147.5 (2–562)	204.7 (2.61–743)	< 0.05*^3^

CRP, CA125, and HE4 concentrations in blood were significantly increased in patients with EOC. Results showed that.

EOC was correlated with age and parity—but not with BMI, smoking, or menopausal status, and that the mean values for CRP, CA125, and HE4 in the EOC group were significantly higher than values in the control group.

*p < 0.05 indicates statistical significance.

^1^ANOVA-test.

^2^Chi-squared test.

^3^Mann–Whitney U test. BMI, body mass index (kg/m^2^).

### CRP, CA125, and HE4 concentrations in blood are significantly increased in patients with EOC

In order to verify whether CRP and tumor markers were significantly increased in patients with EOC, we measured CRP, CA125, and HE4 concentrations in the blood of 91 patients with EOC and 81 controls. As shown in Table 1, in the III/IV-stage, EOC-patient group, the average concentration (range) of CRP was 20.3 (1.8–54) mg/L, CA125 was 162.8 (13.6–691) U/ml, and HE4 was 204.7 (2.61–743) pmol/L. The respective values for the I/II-stage, EOC-patient group were 14.4 (1.6–35) mg/L for CRP, 126 (7.8–427) U/ml for CA125, and 147.5 (2–562) pmol/L for HE4. The benign tumor-patients group exhibited a CRP concentration and range of 4.5 (1.2–14) mg/L, CA125 concentration and range of 21.9 (3.4–122) U/ml, and HE4 of 75.5 (1.5–442) pmol/L. In contrast, in the healthy control group the average CRP concentration and range were 2.8 (0.8–7.3) mg/L, CA125 was 11.5 (3.5–42) U/ml, and HE4 was 53.2 (5.1–252) pmol/L. These results showed that the mean values for CRP, CA125, and HE4 in the EOC group were significantly higher than values in the control group.

### Establishment of diagnostic models and determination of diagnostic capabilities

**Figure 1:**
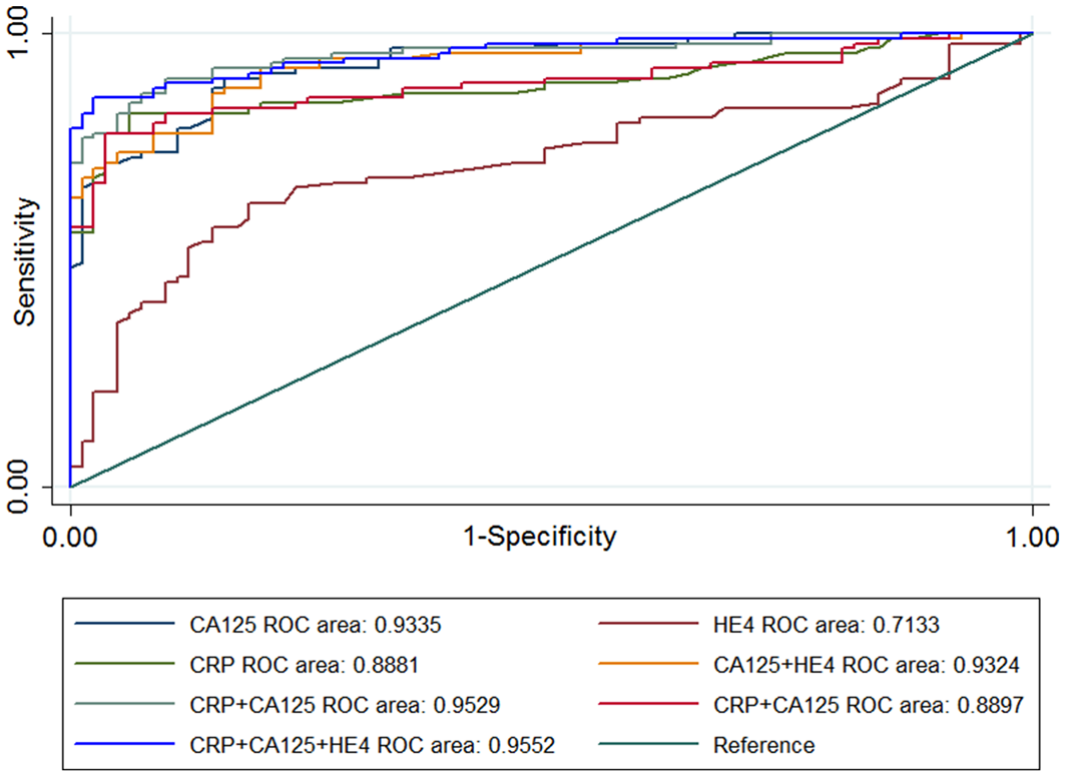
Logistic regression models for evaluating the diagnostic capability of CRP, CA125, and HE4. Results showed that CRP and CA125 exhibited a higher AUC for the diagnosis of EOC relative to HE4. Combining CRP with CA125 or with any of the tumor markers manifested a greater AUC in diagnosing EOC.

To evaluate the diagnostic efficacy of CRP, CA125, and HE4 for EOC, we constructed a diagnostic model for their evaluation so as to compare their diagnostic capabilities. As shown in Fig. 1, the area under the curve (AUC) was used to evaluate the individual and combined diagnostic capabilities of the 3 markers. Table 2 summarizes the AUC, sensitivity, and specificity values of CRP, CA125, and HE4—which were 0.89, 82.4%, and 88.9% for CRP; 0.93, 79.1%, and 88.9% for CA125; and 0.71, 62.6%, and 81.4% for HE4. We evaluated the combined diagnostic capability among the clinical biomarkers, and the results for AUC, sensitivity, and specificity, respectively, were 0.95, 86.8%, and 90.1% for CRP + CA125; 0.89, 82.4%, and 87.6% for CRP + HE4; and 0.95, 87.9%, and 91.3% for CRP + CA125 + HE4. Figure 1 and Table 2 show that CA125 has a higher AUC and specificity for the diagnosis of EOC than do the other tumor markers. The AUC for CRP was second only to CA125, with the specificity the same as for CA125, but the sensitivity was higher than for CA125. Combining CRP with CA125 exhibited augmented the AUC, sensitivity, and specificity, and combining CRP with CA125 and HE4 manifested the highest AUC, sensitivity, and specificity. These findings indicated that CRP is an independent risk factor for EOC, and that CRP combined with CA125 and HE4 can enhance the diagnostic capability for EOC.

**Table 2:** Summary of the AUC, sensitivity, and specificity for CRP, CA125, and HE4 in the diagnosis of EOC.

	AUC	95% CI	Sensitivity (%)	Specificity (%)
CRP	0.89	0.84–0.94	82.4	88.9
CA125	0.93	0.89–0.96	79.1	88.9
HE4	0.71	0.63–0.79	62.6	81.4
CRP + CA125	0.95	0.92–0.98	86.8	90.1
CRP + HE4	0.89	0.84–0.94	82.4	87.6
CA125 + HE4	0.93	0.89–0.96	78.0	87.6
CRP + CA125 + HE4	0.96	0.92–0.98	87.9	91.3

In order to further explore the diagnostic predictive abilities of CRP, CA125, and HE4 on EOC, we calculated the cut-off values for all 3, and their corresponding sensitivities, specificities, positive predictive values (PPV), and negative predictive values (NPV) (Table 3). Our research results showed that CRP specificity (96.3%), PPV (97.2%), and accuracy (86.6%) corresponding to its cut-off value in the diagnosis of EOC were the highest of the indicators, while the sensitivity (83.5%) and NPV (80.0%) corresponding to the cut-off value for CA125 to diagnose EOC were also the highest of the 3. Thus, our research results indicated that CRP was beneficial in the diagnosis of EOC.

**Table 3:** Determination of cut-off values for CRP, CA125, and HE4.

	Cut-off value	Sensitivity at cut-off value (%)	Specificity at cut-off value (%)	PPV at cut-off value (%)	NPV at cut-off value (%)	Accuracy at cut-off value (%)
CRP	9.8 mg/L	78.0	96.3	97.2	77.8	86.6
CA125	26 U/ml	83.5	76.5	80.1	80.0	80.2
HE4	97 pmol/L	53.9	76.6	66.1	64.1	64.5

*PPV* positive predictive value; *NPV* negative predictive value.

### High levels of CRP are associated with the clinicopathologic characteristics of EOC patients

In order to further study the diagnostic efficacy of CRP for EOC, we used logistic regression to find the cut-off value of CRP in EOC. According to the “Youden index,” the “cut-off” value for CRP was 9.8 mg/L—which allowed us to distinguish between EOC patients and control individuals. In our study, 66 of 91 serous EOC patients were above the cut-off value and were therefore classified as high-level CRP groups, while 25 people below the cut-off value were classified as low-level CRP groups. To investigate the correlation between CRP and pathologic parameters, we summarized the correlation between CRP and clinical features of patients with EOC in Table 4. Results showed that among these clinical correlations, high levels of CRP were significantly correlated with tumor size and pathologic staging. The proportion of high-level CRP share was 72% in the group with tumor size > 10 cm, proportion of high-level CRP share in the group with tumor size 5–10 cm was 61%, and the proportion of patients with high-level CRP was 87% in the group with tumor size ≤ 5 cm. The three groups were statistically significant, and the proportion of high-level CRP in the group with tumor size ≤ 5 cm was the highest. The proportion of high-level CRP thus showed statistical significance between International Federation of Gynecology and Obstetrics (FIGO) staging groups. In FIGO stage I and II groups, the proportions of high-level CRP were 83% and 86%, respectively, while in FIGO stage III and IV groups, the proportions of high-level CRP were 56% and 57%, respectively. There were, therefore, statistically significant differences among the 4 groups, with the FIGO stage I/II groups having the highest fractions of high CRP levels. However, the level of CRP among groups was not statistically significant with respect to age, pathologic classification, or pathologic differentiation. These results showing that high-level CRP was correlated with tumor size and stage of EOC indicated that CRP is valuable in the detection of EOC.

**Table 4:** Correlations between concentrations of CRP and clinical parameters of EOC patients.

	Overall (n = 91)	CRP < 9.8 mg/L (n = 25)		CRP > 9.8 mg/L (n = 66)		*p*
		N	%	N	%	
**Age**						0.64^1^
≤ 55	48	12	25	36	75	
> 55	43	13	30	30	70	
**Histopathologic differentiation**						0.72^1^
Poor	25	6	24	19	76	
Moderate	34	11	32	23	68	
Good	32	8	25	24	75	
**FIGO stage**						< 0.05*^1^
I	23	4	17	19	83	
II	29	4	14	25	86	
III	18	8	44	10	56	
IV	21	9	43	12	57	
**Histologic type**						0.99^1^
Serous	42	11	26	31	74	
Mucinous	25	7	28	18	72	
Endometrioid	17	5	29	12	71	
Clear cell	7	2	28	5	72	
**Tumor size**						< 0.05*^1^
≤ 5 cm	38	5	13	33	87	
5–10 cm	46	18	39	28	61	
> 10 cm	7	2	28	5	72	

Results showed that among these clinical correlations, high levels of CRP were significantly correlated with tumor size and pathologic staging. No significant correlation was found between CRP or other clinical parameters. Values of *p < 0.05 were considered statistically significant.

^1^Chi-squared test.

## Discussion

The purpose of this study was to evaluate the diagnostic capability of CRP in EOC, and whether it can be combined with tumor markers to enhance EOC diagnosis. Our results showed that EOC was correlated with age and parity—but not with BMI, smoking, or menopausal status, and that the mean values for CRP, CA125, and HE4 in the EOC group were significantly higher than values in the normal control group. Logistic regression analysis showed that CA125 exhibited the highest diagnostic efficacy for EOC as a single marker, while the sensitivity of CRP was higher than CA125, and the specificity of CRP was equal to that of CA125. The diagnostic efficacy of HE4 was weaker than either CRP or CA125, while the diagnostic capability of the combination of CRP + CA125 + HE4 was the greatest overall. The diagnostic cut-off value with respect to CRP for EOC was 9.8 mg/L, and high-level CRP was correlated with stage and tumor size of EOC. These results indicated that CRP can be used to screen or diagnose EOC in combination with CA125 and HE4, and that high levels CRP demonstrate predictive value with regard to the occurrence of EOC.

Screening methods can increase the efficiency of screening for EOC by increasing sensitivity^22,23^, and multi-marker tests have been shown to improve performance in EOC diagnosis compared to CA125 or HE4 alone^24,25^. Two recent studies showed that combining CA125 and HE4 for determining EOC had a sensitivity of 88% vs. 63% and 78% for either CA125 or HE4 alone, respectively^26,27^, and our results likewise showed that the sensitivity for the combination of CA125 and HE4 was 86.8%. Our results of CA125 (82.4%) and HE4 (62.6%) sensitivities, however, were different from the above results, which may be caused by different disease-entry criteria—as CA125 and HE4 are also upregulated in many other diseases, including uterine fibroids, diverticulitis, endometriosis, liver cirrhosis, normal menstruation, and pregnancy^26–28^. Furthermore, in both of the aforementioned studies, women with ovarian cancer were compared to those with pelvic disease rather than to groups of women that included healthy and benign tumor controls as in our study.

The diagnostic cut-off value for CRP was calculated using the Youden index, and we determined that specificity (96.3%), PPV (97.2%), and accuracy (86.6%) corresponding to the cut-off value for CRP in the diagnosis of EOC were the highest—indicating that CRP provides good diagnostic value in EOC. The cut-off value for CRP was 9.8 mg/L, and we classified CRP concentrations above 9.8 mg/L as high-level CRP. Our statistical results showed that high-level CRP was correlated with tumor stage and tumor size, but showed no correlation with age, pathologic type, or pathologic differentiation. We also reported that the earlier the stage, the smaller the tumor, and the higher the proportion of high-level CRP were of great significance in the diagnosis of EOC. In EOC, CRP has been consistently associated with risk**,** with a recent meta-analysis^29^ noting that EOC risk rises with increasing CRP concentration, and the risk of EOC in women with CRP concentrations greater than 10 mg/L was more than doubled. Our cut-off value of CRP for EOC was 9.8 mg/L, which is consistent with the results of previous studies that showed a high-risk predictive value of CRP for EOC at 10 mg/L^30,31^. However, small sample size leads to imprecision in estimates of performance, and therefore sample sizes should be increased in future research. In the actual diagnostic process, tumor patients might be combined with individuals displaying other cardiovascular, cerebrovascular, inflammatory, or autoimmune diseases^14^, and thus the diagnostic value of CRP for EOC may be limited to an extent. In addition, as tumors progress, disease complications and treatment with CRP increase or decrease commensurately. For example, a middle-aged female patient without other diseases should be alerted to the occurrence of EOC when blood CRP concentrations long term are higher than 9.8 mg/L.

Tumors can induce non-specific inflammation through the tumor microenvironment, and this contributes to the release of pro-inflammatory factors that can induce the secretion by liver cells of CRP into blood^32^. Investigators previously found that levels of CRP were significantly elevated in blood of patients with tumors, and that the increase was correlated with tumor differentiation, tumor metastasis, and postoperative survival rate^33–37^. We demonstrated that EOC was correlated with parity, and that lower parity was associated with a higher risk of EOC. Intriguingly, some studies have shown that EOC may be related to ovulation, as ovarian epithelium can be damaged and repaired during the ovulatory process, which may cause the malignant transformation of ovarian epithelium^38,39^. Therefore, women with higher parity may show fewer ovulations during pregnancy and lactation, and thus have a reduced risk of EOC. There is no evidence that CRP promotes the development of cancer, but the increase in circulating CRP with the onset of EOC may be caused by inflammation in the tumor microenvironment^16^, and inflammation is believed to exert carcinogenic actions^40^. Inflammation directly participates in tumor development by generating toxic oxidants and biologically active substances, and these may damage DNA and proteins, thereby increasing the possibility of mutagenesis^30^. In the microenvironment surrounding tumor formation, ovarian surface epithelium may therefore be continuously exposed to an inflammatory environment in which CRP upregulation follows activation of the proinflammatory factors interleukin-6 (IL-6) and tumor necrosis factor-alpha (TNF-α)^40^. Our results thus provide evidence that CRP can be used to predict the occurrence of EOC.

Our results showed that the combined use of CRP with CA125 and HE4 improved the diagnostic capability toward EOC. CRP (> 9.8 mg/L) also exhibited a high value in the diagnosis of EOC and can be used as a diagnostic marker for this cancer—especially in the absence of other diseases. We thus provided new evidence for the efficacy of CRP in diagnosing EOC. Given that CRP is easily detectable in the serum—and that CRP is a highly sensitive marker of inflammation—additional research is necessary to prove that CRP is a good biomarker of EOC risk.

## Methods

### Specimens and patients

Clinical parameters from 91 EOC patients and 81 samples from control groups (a mix of patients with benign ovarian tumors, including simple ovarian cyst or benign cystadenoma and healthy candidates) were obtained from the Affiliated Hospital of Qingdao University and Qingdao Haici Hospital between February 2017 and December 2019. Informed consent was received from all patients involved in this study, and the study was approved by the Ethics Committees of the Affiliated Hospital of Qingdao University and Qingdao Haici Hospital. All experiments were performed in accordance with the relevant guidelines of a protocol approved by the Institutional Review Board from Qingdao Haici Hospital and the Affiliated Hospital of Qingdao University. None of the patients had received tumor treatment prior to confirmed pathologic diagnosis. All blood samples from EOC patients and participants were collected after initial diagnosis and before treatment or surgical operation. The following inclusion criteria were developed to recruit participants to the study: I) tumor without surgical operation and tumor confirmed by pathology postoperatively, II) white blood cell count and proportions of neutrophils tested for the exclusion of bacterial infections, and III) exclusion of cardiovascular and cerebrovascular diseases, traumatic disease, immune diseases, other tumors, primary disease of liver or kidney, pregnancy or lactating, or participating in other clinical trials.

### Collection and detection of serum biomarkers

Collected serum specimens were tested immediately or preserved at -20ºC for testing within 20 days. We measured serum levels of hs-CRP (high sensitivity-CRP), CA125, and HE4 using the Roche Cobas 8000 system (Roche, Switzerland) at the Qingdao Third People’s Hospital.

### Statistical analysis

We performed all statistical analyses using Stata 12.0 software. Statistical significances for CRP, CA125, and HE4 of participants were calculated using the *Mann–Whitney test*. Comparisons of age and BMI among groups were determined using 1*-way ANOVA*, and rates of groups were compared using *the Chi-squared test*. We constructed a *logistic regression* model utilizing CRP or conventional tumor markers for the diagnosis of EOC. A receiver operating characteristic curve (ROC) was calculated to evaluate the diagnostic capabilities of the biomarkers. Values of *p* < 0.05 were considered statistically significant.

We decided our cut-off value using the “Youden index”: the calculation of the Youden index is the sum of the sensitivity and specificity minus one, the detection value corresponding to the maximal Youden index was then designated as the cut-off value. According to the Youden index, the cut-off value for CRP that can distinguish EOC patients from non-EOC patients in our study was 9.8 mg/L.

## Data Availability

All authors agreed to make materials, data, and associated protocols available to readers without undue qualifications in material transfer agreements.
